# Virulence Characteristics of *Klebsiella* and Clinical Manifestations of *K. pneumoniae* Bloodstream Infections

**DOI:** 10.3201/eid1307.070187

**Published:** 2007-07

**Authors:** Victor L. Yu, Dennis S. Hansen, Wen Chien Ko, Asia Sagnimeni, Keith P. Klugman, Anne von Gottberg, Herman Goossens, Marilyn M. Wagener, Vicente J. Benedi, the International Klebsiella Study Group

**Affiliations:** * University of Pittsburgh, Pittsburgh, Pennsylvania, USA; † Statens Serum Institut, Copenhagen, Denmark; ‡ National Cheng Kung University Medical College, Tainan, Taiwan, Republic of China; § Emory University, Atlanta, Georgia, USA; ¶ South African Institute of Medical Research, Johannesburg, South Africa; # University Hospital, Antwerp, Belgium; ** Universidad de las Islas Baleares, Palma de Mallorca, Spain; 1 Deceased.; 2 The International Klebsiella Study Group comprises the previously named authors plus Jose Maria Casellas, Gordon Trenholme, Joseph McCormack, Sunita Mohapatra, and Lutfiye Mulazimoglu.

**Keywords:** *Klebsiella pneumoniae*, gram-negative bacteremia, virulence, epidemiology, research

## Abstract

We studied 455 consecutive episodes of *Klebsiella pneumoniae* bacteremia occurring in 7 countries. Community-acquired pneumonia and an invasive syndrome of liver abscess, meningitis, or endophthalmitis occurred only in Taiwan and South Africa. Infections by K1 and K2 capsular serotype, the mucoid phenotype, and aerobactin production were important determinants of virulence. The mucoid phenotype was seen in 94% of isolates in patients with community-acquired pneumonia and in 100% of isolates that caused the invasive syndrome in Taiwan and South Africa, compared with only 2% of isolates elsewhere. Mortality of mice injected with mucoid strains (69%) was strikingly higher than that occurring in mice injected with nonmucoid strains (3%, p<0.001). Differences in clinical features of bacteremic infection with *K. pneumoniae* are due to the virulence factors expressed by the organism.

## Abstract 2

Differences in clinical manifestations are due to virulence factors expressed by the organism.

In the past decade, geographic differences have been recognized in the spectrum of disease caused by *Klebsiella pneumoniae*. These differences include a preponderance of severe invasive disease in Taiwan and other parts of Asia (*1*–*8*). A characteristic syndrome has emerged in which liver abscess is accompanied by *K. pneumoniae* bacteremia and sometimes by endophthalmitis or meningitis. This is typically a community-acquired infection that occurs in patients with diabetes mellitus. Reports of this syndrome from North America, Europe, and Australia are uncommon (*2*).

Additionally, *K. pneumoniae* has long been recognized as a possible cause of community-acquired pneumonia. Over the past 2 decades, *K. pneumoniae* has been an exceedingly rare cause of community-acquired pneumonia in North America, Europe, and Australia (*2*,*9*,*10*). Yet, it remains an important cause of severe community-acquired pneumonia in Asia and Africa (*11*–*15*). In these regions, patients also have classic risk factor of alcoholism (*2*).

We have completed a prospective study of 455 patients from 7 countries with *K. pneumoniae* bacteremia (*2*). We found that although nosocomial infections with *K. pneumoniae* occurred worldwide, some manifestations of community-acquired infection (namely, liver abscess and community-acquired pneumonia) were geographically restricted. These manifestations of disease occurred almost exclusively in Taiwan and South Africa (*2*). Potential explanations for these geographic differences in clinical manifestations include host factors such as rates of diabetes mellitus, alcoholism, access to healthcare, and socioeconomic factors.

Another explanation for these differences is related to the organism. In this study, we performed capsular serotyping, determined the presence of mucoid phenotype and aerobactin production, and assessed lethality in a murine model and correlated these in vitro and in vivo results with the clinical manifestation of patients with *K. pneumoniae* bloodstream infections. Our aim was to determine whether the different manifestations of infection occurring in different geographic regions could be correlated with differences in organism characteristics.

## Methods

### Study Design

A prospective, observational study of consecutive, sequentially encountered patients with *K. pneumoniae* bacteremia was conducted in 12 hospitals in the United States, Taiwan, Australia, South Africa, Turkey, Belgium, and Argentina. No patients were excluded from analysis. The study period was January 1, 1996, to December 31, 1997. Patients >16 years of age with positive blood cultures for *K. pneumoniae* were enrolled and completed a 188-item study form. Patients were followed up for 1 month after the onset of bacteremia to assess clinical outcome, including deaths and infectious complications. The study was observational in that administration of antimicrobial agents and other therapeutic management were controlled by the patient’s physician, not the investigators. The study was approved by institutional review boards as required by local hospital policy.

### Definitions

Terms were defined a priori (that is, before data analysis). Community-acquired bacteremia was defined as a positive blood culture taken on admission or within 48 hours of admission. Site of infection accompanying the bacteremia was determined as pneumonia, urinary tract infection, meningitis, incisional wound infection, other soft tissue infection, intraabdominal infection, and primary bloodstream infection by using Centers for Disease Control and Prevention definitions (*16*). “Invasive” infections accompanying *K. pneumoniae* bacteremia were further defined as liver abscess, meningitis, or endophthalmitis. Liver abscess was defined by the coexistence of blood cultures positive for *K. pneumoniae* and evidence of an intrahepatic abscess cavity by ultrasonography or computed tomography. Meningitis was defined as culture of *K. pneumoniae* from the cerebrospinal fluid. Endophthalmitis was defined as decreased visual acuity, pain, hypopyon, or severe anterior uveitis concurrent with *K. pneumoniae* bacteremia in a patient.

### Microbiology

Blood culture isolates of *K. pneumoniae* were sent by the participating hospitals on nutrient agar slants to the Special Pathogens Laboratory in Pittsburgh. There, the identity of each isolate as *K. pneumoniae* was confirmed by using the Vitek GNI system (bioMérieux Vitek, Hazelwood, MO, USA).

**Figure 1:**
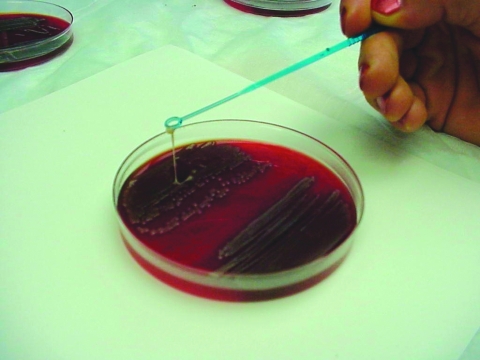
Mucoid phenotype of *Klebsiella pneumoniae.* When colonies were touched with a loop and the loop lifted vertically from the surface of the agar plate, mucoid isolates adhered to the loop as it was lifted from the plate. (Figure first presented at the 36th annual conference of the Infectious Diseases Society of America, Denver, Colorado, USA, 1998.)

The isolates were classified phenotypically as mucoid or nonmucoid. Colonies were touched with a loop; the loop was then lifted vertically from the surface of the agar plate. Mucoid phenotype was defined as being present when a stringlike growth was observed to attach to the loop as it was lifted from the plate (Figure 1). Presence of the *rmpA* gene (rmp = regulator of the mucoid phenotype) was sought by DNA dot blot hybridization by using a 640-bp probe (position 478–1117 of the *rmpA* gene; accession no. X17518). The probe was produced by direct digoxigenin (DIG)-labeled PCR by using the PCR DIG probe synthesis kit (Roche Diagnostics, Basel, Switzerland) and the primers Kleb_MP_F1 (5′-GAG CAA AGT TAC TGT TTC TAT GGA-3′) and Kleb_MP-R1 (5′-TGA GCC ATC TTT CAT CAA CC-3′) on the *K. pneumoniae* strain B 5055. Dot blot hybridization was performed according to the manufacturer’s protocol on Hybond N+ nylon membranes (Amersham, Pharmacia Biotech, Piscataway, NJ, USA), and the hybridized probe was visualized by using the DIG nucleic acid detection kit (Roche Diagnostics).

Capsular K serotyping was performed at the World Health Organization International *Escherichia* and *Klebsiella* Reference Centre (Copenhagen, Denmark) by using standard methods. In brief, K-typing was conducted by counter current immunoelectrophoresis (CCIE) with a modified version of the method described by Palfreyman (*17*). An extract was used as antigen instead of a whole cell suspension; the extract use was a modification because it was only heated once for 1 h at 100°C before centrifugation (*18*). All isolates with negative or doubtful reactions in CCIE were investigated by the classic Quellung technique, and K-type nontypeable isolates were investigated for the presence (K+) or absence (K–) of a visible capsule by wet mount microscopy with India ink. Lipopolysaccharide O typing was performed by a previously described inhibition ELISA (*19*).

Aerobactin production was demonstrated by a cross-feeding bioassay that used *Escherichia coli* strain LG 1522 (*20*). The clinical isolates were grown overnight in M9 broth containing the iron chelator 2-2′ dipyridyl. Strains were spotted onto hardened dipyridyl minimal agar plates. After 18 hours’ incubation at 37°C, satellite growth of the indicator strain LG 1522 around the spots indicated aerobactin production.

### Lethality in Mice

A standard inoculum of 1–2 × 10^7^ bacteria in the logarithmic phase of growth from blood culture isolates from each study site was injected intravenously into the tail vein of C57/BL6J black, female mice, 8–12 weeks old. Two mice (Jackson Laboratories, Bar Harbor, ME, USA) were inoculated with each strain. Mortality of the mice was observed at 24 hours postinjection. The animal experiments were approved by the Institutional Review Board of the Veterans Affairs Medical Center, Pittsburgh, Pennsylvania, USA.

### Pulsed-Field Gel Electrophoresis (PFGE)

The genotypic relationships of *K. pneumoniae* bloodstream isolates were determined by using PFGE. PFGE was performed by means of the CHEF-DR II system (Bio-Rad, Richmond, CA, USA) with use of the restriction endonuclease Xba I (New England Biolabs, Beverly, MA, USA). DNA was subjected to electrophoresis for 22 hours at 14°C in a 1% agarose gel at 6 V/cm with a linear gradient pulse time of 5–35 seconds. The gels were analyzed by using the Gel Doc 2000 software (Bio-Rad).

### Statistics

Patient demographics and laboratory data were entered into PROPHET Statistics version 6.0 (AbTech Corporation, Charlottesville, VA, USA). The χ^2^ or Fisher test was used to compare categorical variables. Continuous variables were compared by using the *t* test or the Mann-Whitney test. Multivariate analysis was used to determine which risk factors for mouse lethality by univariate analysis were independently significant.

## Results

### Serotypes of *K. pneumoniae* Bacteremic Strains

During the study period, 455 episodes of *K. pneumoniae* bloodstream infection occurred; 141 community-acquired bloodstream isolates were available and were tested for K serotype. Three isolates were not encapsulated, and 20 were nontypeable. Forty-seven different capsular serotypes were found in the remaining strains; K1 (16%; 23/141), K2 (11%; 16/141), and K54 (9%; 12/141) were the most common serotypes.

One hundred percent (23/23) of K1 serotype strains, 94% (15/16) of K2 serotype strains, and 100% (12/12) of K54 serotype strains were from Taiwan or South Africa. Forty-seven percent (23/49) of isolates from patients with community-acquired pneumonia and 50% (7/14) of isolates from patients with invasive syndromes possessed the K1 or K2 serotype. In comparison, 12% (9/78) isolates from patients with other manifestations of community-acquired *K. pneumoniae* bacteremia had the K1 or K2 serotype (p<0.001).

Seventy-three randomly chosen hospital-acquired bloodstream isolates from the same multinational study were tested for K serotype. One isolate was not encapsulated, and 5 isolates were nontypeable. In contrast to the predominance of K1 serotypes in community-acquired infections, 1% (1/75) of hospital-acquired isolates were K1 (p = 0.0015). The percentage of K2 serotypes in hospital acquired isolates (9%, 7/75) was similar to that observed in community-acquired isolates (11%, 16/141) (p = 0.76). The hospital-acquired K1 isolate was from Taiwan; 4 hospital-acquired K2 isolates were from Africa, 2 were from the United States, and 1 was from Taiwan. In contrast to the predominance of K1 and K2 serotypes in community-acquired pneumonia (47%, 23/49 isolates), these serotypes were significantly less likely to occur in nosocomial pneumonia (11%, 2/18 cases; p = 0.022).

### Relationship between Capsular Serotype and Lethality in Mice

When community-acquired strains only were examined, highest lethality was observed with strains of K1 serotype (mouse deaths 65%) and K2 serotype (mouse deaths 81%). In contrast, lethality for community-acquired strains of other serotypes was significantly lower (23% died; p<0.001). The proportion of deaths in mice injected with community-acquired strains of serotype K54 was 9%.

When hospital-acquired strains were examined, mouse mortality was only 8%. The only lethal strains were K1 and K17 serotypes. None of the 7 hospital-acquired strains of serotype K2 proved lethal to mice.

When both community- and hospital-acquired strains were assessed together, lethality was significantly higher for strains of K1 (mouse deaths 67%) and K2 serotype (mouse deaths 59%) than for strains of other serotype (mouse deaths 20%) (p<0.001). The lethality of all strains of serotype 54 was 12.5%.

### Lipopolysaccharide O Typing

Of 195 isolates which underwent O typing, 30% (58/195) were group O1, 17% (34/195) were O2, 8% (16/195) were O3, 2% (3/195) were O4, 3% (6/195) were O5, 1% (2/195) were O2ac, 7% (13/195) were O–, and 32% (63/195) were O+ but not typable. No strains of O group O3, O4, O5, O2ac or O– were lethal to mice. In contrast, 21% (12/58) of O1 strains, 26% (9/34) of O2 strains, and 37% (23/63) of nontypeable O+ strains were lethal to mice.

### Relationship between Mucoid Phenotype and Type of Infection

The mucoid phenotype was observed in 93% (13/14) of strains from patients with invasive disease, in 67% (33/49) of strains from patients with community-acquired pneumonia, and in 28% (22/78) of strains from patients with other sites of community-acquired infection (p<0.001). Mucoid strains of community-acquired bacteremic *K. pneumoniae* were exclusively found in Taiwan and South Africa; 56% (45/80) and 60% (21/35) of Taiwanese and African strains, respectively, were mucoid, whereas no mucoid strains were detected in community-acquired bacteremic strains from other countries (Table 1). In Taiwan and South Africa, mucoid strains predominated in community-acquired strains (57%, 66/115) compared with hospital-acquired strains (18%, 7/38) (Table 2).

**Table 1:** Disease type by virulence factor*

Source of organism and type of infection	K1 or K2 serotype, %	Mucoid phenotype, %	Aerobactin producer, %	Mouse mortality rate, %†
Taiwan				
Community-acquired	28 (22/80)	56 (45/80)	54 (43/80)	46
Hospital-acquired	17 (2/12)	25 (3/12)	33 (4/12)	29
South Africa				
Community-acquired	46 (16/35)	60 (21/35)	63 (22/35)	52
Hospital-acquired	16 (4/25)	16 (4/25)	8 (2/25)	0
Rest of world				
Community-acquired	4 (1/26)	0 (0/26)	4 (1.26)	0
Hospital-acquired	5 (2/36)	8 (3/36)	8 (3/36)	0

*Note the virtual absence of putative virulence factors in strains from the rest of the world, other than Taiwan and South Africa. See Results for p values. †2 mice were tested for each available strain.

**Table 2:** Strain source and virulence factors, Taiwan and South Africa*

Infection type	K1 or K2 serotype, %	Mucoid phenotype, %	Aerobactin producer, %	Mouse mortality rate, %†
Community-acquired pneumonia	49 (23/47)	68 (32/47)	66 (31/47)	47
Invasive syndrome	54 (7/13)	100 (13/13)	85 (11/13)	82
Other community-acquired	15 (8/55)	38 (21/55)	42 (23/55)	36
Hospital-acquired	16 (6/38)	18 (7/37)	16 (6/38)	7

*Note that strains from patients in Taiwan and South Africa with community-acquired pneumonia or the invasive syndrome (liver abscess, endophthalmitis, meningitis) were more likely to have the putative virulence factors than hospital-acquired strains. See Results for p values. †2 mice were tested for each available strain.

In Taiwan and South Africa, community-acquired pneumonia was due to mucoid strains of *K. pneumoniae* in younger patients with no serious underlying disease, while nonmucoid strains predominated in older patients and those with serious underlying disease (Table 3). In both countries combined, 94% (29/31) strains from community-acquired pneumonia patients with no serious underlying disease had the mucoid phenotype compared to 19% (3/16) strains from patients with serious underlying disease (p<0.001). All isolates (13/13) from patients in Taiwan or South Africa with the invasive syndrome of liver abscess, meningitis, or endophthalmitis had the mucoid phenotype (Table 4).

**Table 3:** Underlying disease and virulence factors in community-acquired *K. pneumoniae* pneumonia*

Country/condition	K1 or K2 serotype, %	Mucoid phenotype, %	Aerobactin production, %	Mouse mortality rate, %†
South Africa	50 (12/24)	75 (18/24)	67 (16/24)	58
No underlying disease	63 (12/19)	89 (17/19)	79 (15/19)	78
Underlying disease‡	0 (0/5)	20 (1/5)	20 (1/5)	0
Taiwan	48 (11/43)	65 (15/23)	57 (13/23)	35
No underlying disease	75 (9/12)	100 (12/12)	83 (10/12)	50
Underlying disease‡	18 (2/11)	18 (2/11)	27 (3/11)	18

*Note that patients with no underlying disease were more likely to be infected by strains with the putative risk factors than were elderly patients or patients with serious underlying disease. See Results for p values. †2 mice were tested for each available strain. ‡Underlying disease was defined as presence of end-stage liver or renal failure, metastatic malignancy, neutropenia, or age >70.

**Table 4:** Mucoid strains in patients with liver abscess, endophthalmitis, or meningitis associated with community-acquired *Klebsiella pneumoniae* bacteremia*

Country	K1 or K2 serotype, %	Mucoid phenotype, %	Aerobactin producer, %	Mouse mortality rate, %†
Taiwan	50 (6/12)	100 (12/12)	85 (10/12)	81
South Africa	100 (1/1)	100 (1/1)	100 (1/1)	100

*Mucoid strains are highly lethal to mice. †2 mice were tested for each available strain.

The proportion of deaths in mice injected with mucoid strains (69% of mice died) was strikingly higher than that occurring in mice injected with nonmucoid strains (3% mice died) (p<0.001). There was no association between human deaths and the presence of a mucoid strain (39% died) or a nonmucoid strain (35% died) (p>0.20). In a multivariate model, increased severity of illness score when first evaluated (p = 0.0001), but not infection with a mucoid strain, country of origin, or history of alcoholism (p>0.20 for all) was associated with human deaths.

### Association between Phenotypic Evidence of Mucoidity and Presence of *rmpA* Gene

Phenotypic evidence of mucoidity as judged by the definition in the methods section (“a string-like growth observed to attach to the loop as it was lifted from the plate”) (Figure 1) was highly correlated with the presence of the *rmpA* gene. Of 77 mucoid isolates, 86% (66/77) were *rmpA* gene positive, and 14% (11/77) were *rmpA* gene negative. Of 137 nonmucoid isolates, 93% (128/137) were negative for the *rmpA* gene and 7% (9/137) were *rmpA* positive.

### Relationship between Aerobactin Production and Type of Infection

The presence of the *rmpA* gene and phenotypic evidence of aerobactin production were closely correlated. Ninety-six percent of *rmpA* gene–positive isolates were aerobactin producers; aerobactin was produced by 2% of isolates that were *rmpA* gene–negative.

Associations between aerobactin production and type of infection were similar to those between the mucoid phenotype and type of infection. Only 6% (4/62) strains from patients in countries other than Taiwan and South Africa were aerobactin producers. In Taiwan and South Africa, 66% of patients with community-acquired pneumonia and 85% of patients with the invasive syndrome had aerobactin-producing strains, in comparison with 42% of patients with other community-acquired infections and 16% of patients with hospital-acquired strains (Table 2).

### Relationship between Mucoid Phenotype, Capsular Serotype, and Lethality in Mice

When both community-acquired and hospital-acquired strains were considered together, 77% (36/47) of isolates of serotypes K1 and K2 were found to be mucoid. Of the other 45 serotypes, 24% (40/167) were mucoid (p<0.001).

However, none of the mice inoculated with nonmucoid K1 or K2 serotype strains died, compared with 81% of mice inoculated with mucoid K1 or K2 serotype strains (p<0.001). Just 4% of mice inoculated with nonmucoid strains of serotypes other than K1 or K2 died, compared with 44% mice inoculated with mucoid organisms of serotypes other than K1 or K2 (p<0.001).

When the parameters of mucoid phenotype, serotypes K1 and K2, and country of origin were assessed in the multivariate model of lethality to mice, mucoid phenotype was strongly associated with the death of mice (p = 0.001). Presence of serotypes K1 and K2 approached statistical significance (p = 0.05).

### PFGE

**Figure 2:**
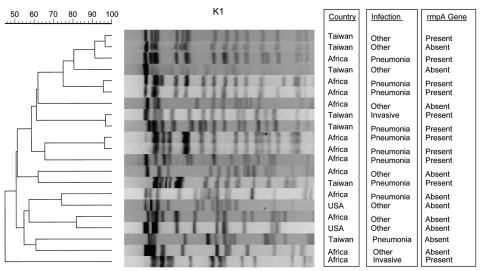
Pulsed-field gel electrophoresis of bacteremic *Klebsiella pneumoniae* isolates of serotype K1.

**Figure 3:**
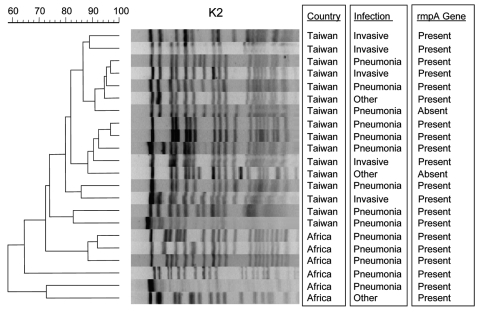
Pulsed-field gel electrophoresis of bacteremic *Klebsiella pneumoniae* isolates of serotype K2.

PFGE was performed on strains of the same serotype. Dendrograms of organisms of serotype K1 are shown in Figure 2 and dendrograms of serotype K2 in Figure 3.

## Discussion

We have been able to evaluate geographic differences in community-acquired *K. pneumoniae* infections by studying consecutive patients with community-acquired *K. pneumoniae* bacteremia from 7 different countries during the same period. It could be hypothesized that patient characteristics are primarily responsible for these differences. For example, genetic predilections (susceptibility of Asians to liver abscess), underlying diseases (for example, higher prevalence of chronic hepatitis B virus infection in Taiwan), social factors (different foods or cultural practices), and economic factors (for example, access to healthcare, antimicrobial drug usage) may be responsible for the different manifestations of serious *K. pneumoniae* infection observed in different regions. Despite these other possibilities, our experimental studies suggest that the differences in clinical features arise from differences in the virulence of individual microorganisms.

In particular, we found that strains with K1 or K2 serotype, strains with a mucoid phenotype, and strains that are capable of aerobactin production are rarely found to cause substantial infection in patients from study hospitals outside Taiwan and South Africa. Strains with such virulence characteristics were more likely to cause community-acquired infections than hospital-acquired infections. When strains with these virulence characteristics were inoculated into mice, deaths exceeded 80% compared with mortality rates of <5% in mice inoculated with strains lacking these characteristics.

Additionally, by PFGE, we found genetically related strains possessing all 3 virulence characteristics (Figures 2, 3). Taiwanese investigators have debated whether *K. pneumoniae* strains that cause liver abscess in Taiwan are clonally related (*1*,*4*,*6*,*7*,*21*). Genetic relatedness in *K. pneumoniae* strains that cause community-acquired pneumonia has not been previously described. However, we have found that genotypically related organisms were responsible for bacteremic community-acquired pneumonia due to *K. pneumoniae* and sometimes both pneumonia and liver abscess or meningitis. Whether *K. pneumoniae* is spread from person to person, whether related strains are acquired from common sources, or whether virulent strains arise from a common ancestor remains to be determined.

Much prominence has been placed in the past on the role of capsule in the pathogenesis of *K. pneumoniae* infections. Capsular types K1 and K2 have been regarded as particularly virulent (*22*). Taiwanese researchers have found that serotype K1 is frequently associated with community-acquired *K. pneumoniae* bacteremia (*3*,*23*). We have also confirmed that serotypes K1 and K2 occur more frequently in isolates from community-acquired infections in Taiwan and South Africa than from hospital-acquired isolates in these countries or elsewhere (Table 1).

The degree of virulence conferred by a particular K antigen may be related to the mannose content of the capsular polysaccharide. Capsular types with high virulence in animal models (for example, K2) lack mannose-α-2/3-mannose structures found in capsular types of lower virulence (*24*). The mannose-α-2/3-mannose structures are recognized by a surface lectin of macrophages, which mediate complement and antibody-independent phagocytosis. Strains that lack these sequences (for example, those with the K2 antigen) may not be recognized by macrophages, and hence phagocytosis may not take place. Furthermore, surfactant protein A (the main protein component of lung surfactant) enhances the phagocytosis by alveolar macrophages of strains that bear mannose-α-2/3-mannose structures in their capsule, but not strains which lack the mannose structure (*25*).

Some strains belonging to the K2 serotype are not as virulent as others (*26*). Thus, factors other than capsule may also be important for virulence. Although previous authors did not find any markers for these differences (*26*), we found that mucoid strains of K1 or K2 serotype were more virulent to mice than nonmucoid strains of the same serotype. No mouse inoculated with nonmucoid K1 or K2 serotype strains died, compared to 81% mice inoculated with mucoid K1 or K2 serotype strains (p<0.001). Multivariate analysis of variables related to mouse mortality rates showed that mucoidity was more closely associated with death than was capsular serotype. Contrary to popular belief, the biochemical nature of the mucoid phenotype may be unrelated to capsular polysaccharide but rather related to extracapsular polysaccharide (*27*). A previous study has shown that the mucoid phenotype may be due to a gene designated *rmpA* (regulator of mucoid phenotype) (*28*). In another mouse model, a mutant carrying this gene was 1,000-fold more virulent than an isolate without the gene. Extracellular polysaccharides may protect mucoid strains of *K. pneumoniae* from phagocytosis by neutrophils and from serum killing by complements (*27*).

We found that the mucoid phenotype frequently coexists with aerobactin production. The growth of bacteria in host tissues is limited not only by host defense mechanisms but also by its supply of available iron. The supply of free iron in the host milieu may be extremely low; many bacteria attempt to secure their supply of iron in the host by secreting high-affinity iron chelators called siderophores. Aerobactin is a hydroxamate-type siderophore occasionally found in *Klebsiella* strains. *K. pneumoniae* strains that produce aerobactin were more virulent in our mouse model, whereas strains not producing this siderophore were less likely to be; additionally, patients with severe community-acquired infection were more likely to be infected by aerobactin-producing strains. In another mouse model, transfer of a recombinant plasmid harboring the genes for aerobactin and its receptor enhanced the virulence of an otherwise avirulent strain by 100-fold (*29*). The strong association found in this study between mucoid phenotype (*rmpA* positive isolates) and aerobactin production suggests that the 2 virulence characteristics might be genetically coupled on a large virulence plasmid, as has previously been demonstrated (*28*). We have not yet determined which of these 2 virulence factors is more important.

Strains harboring these virulence factors appear to be more frequent in certain geographic regions, and this may explain geographic differences in manifestation of community-acquired *Klebsiella* infections. The evolutionary genetics of *K. pneumoniae* have never been explored. To our knowledge, we have been the first to find clones bearing multiple virulence characteristics that are responsible for life-threatening community-acquired *K. pneumoniae* pneumonia in otherwise healthy persons. Investigation into the mechanisms of virulence in *K. pneumoniae* could lead to preventive measures (such as vaccination) in high-risk parts of the world.
